# STATs: An Old Story, Yet Mesmerizing

**DOI:** 10.22074/cellj.2015.1

**Published:** 2015-10-07

**Authors:** Saeid Abroun, Najmaldin Saki, Mohammad Ahmadvand, Farahnaz Asghari, Fatemeh Salari, Fakher Rahim

**Affiliations:** 1 Department of Hematology, Faculty of Medical Sciences, Tarbiat Modares University, Tehran, Iran; 2 Health Research Institute, Research Center of Thalassemia and Hemoglobinopathy, Jundishapur University of Medical Sciences, Ahvaz, Iran; 3 Department of Medicine II, Division of Gastroenterology, University of Rostock, E.Heydemann-Strasse 6, Rostock, Germany; 4 Health Research Institute, Hearing Research Center, Ahvaz Jundishapur University of Medical Sciences, Ahvaz, Iran

**Keywords:** JAK, STAT, Signaling Pathways, Malignancy, miRNA

## Abstract

Signal transducers and activators of transcription (STATs) are cytoplasmic transcription factors that have a key role in cell fate. STATs, a protein family comprised of seven members, are proteins which are latent cytoplasmic transcription factors that convey signals from the cell surface to the nucleus through activation by cytokines and growth factors. The signaling pathways have diverse biological functions that include roles in cell differentiation, proliferation, development, apoptosis, and inflammation which place them at the center of a very active area of research. In this review we explain Janus kinase (JAK)/STAT signaling and focus on STAT3, which is transient from cytoplasm to nucleus after phosphorylation. This procedure controls fundamental biological processes by regulating nuclear genes controlling cell proliferation, survival, and development. In some hematopoietic disorders and cancers, overexpression and activation of STAT3 result in high proliferation, suppression of cell differentiation and inhibition of cell maturation. This article focuses on STAT3 and its role in malignancy, in addition to the role of microRNAs (miRNAs) on STAT3 activation in certain cancers.

## Introduction

Signal transducers and activators of transcriptions (STATs), originally discovered as DNA-binding proteins, mediate interferon-dependent gene expression (1-3). STATs are latent transcription factors activated by extracellular signaling ligands such as cytokines, growth factors and hormones (4, 5). These transducers become activated in the cytoplasm by Janus kinase (JAK), a family of tyrosine kinases (TKs). These signaling pathways have diverse biological functions which include roles in cell differentiation, proliferation, development, apoptosis, and inflammation that make them a very active area of research (6). In contrast to the restricted role of STATs 1, 2, 4 and 6 (Table 1), STAT3 and STAT5 have broader functions in disease resistance to treatments. In the JAK/ STAT pathway, STAT3 has a broad role in cell function; its aberration contributes to excessive cell growth and proliferation. Interestingly, elevated levels of STAT3 have been observed in many human cancers and cancer cell lines (7). This review article presents an overview of the JAK/STAT pathway followed by an investigation of the role of STAT3 under normal and malignant conditions. Finally, we discuss the regulatory role of microRNAs (miRNAs) on STAT3 expression, as a new hot topic in therapeutics.

**Table 1:** Cytokines induce the activation of Janus kinase (JAKs) and signal transducers and activators of transcription (STATs) proteins http://flipper.diff.org/app/pathways/info/1565


Cytokines	Interferons			gp130 family			βc family	γc family			Homodimeric receptors			GPCRs	
	Type I IFNα/β	Type II IFN γ	Type III IL-10	IL-6, 11 LIF, G-CSF OSM	IL-12	Leptin	IL-3, IL-5 GM-CSF	IL-2, 7 IL-9, 15	IL-4	IL-13	GH	EPO Prl	TPO	Angi.	Serot.

JAK1	*	*	*	*				*	*	*					
JAK2		*		*	*	*	*				*	*	*	*	*
JAK3								*	*						
TYK2	*		*	*	*					*				*	
STAT1	*	*	*	*				*			*		*	*	
STAT2	*													*	
STAT3	*		*	*		*		*			*		*	*	*
STAT4					*			*							
STAT5a/b	*	*		*			*	*			*	*	*	*	
STAT6									*	*					


IFN; Interferon, IL; Interleukin, βc family; Common beta receptor subunit, γc family; Common gamma receptor subunit, G-CSF; Granulo- cyte colony stimulating factor, GH; Growth hormone, GM-CSF; Granulocyte macrophage colony-stimulating factor, EPO; Erythropoietin, TPO; Thrombopoietin, Prl; Prolactin, Angi.; Angiotensin, Serot.; Serotonin ,*; Activation by cytokine, LIF; Leukemia inhibitory factor, GP- CRs; G-protein-coupled receptors and TYK2; Tyrosine kinase 2.

### Overview of JAK family structure and function

In contrast to other TK families, the JAK family is small. There are only four known mammalian JAKs-JAK1, JAK2, JAK3, and TYK2 that have been identified in the early 1990s by techniques that capitalized on homology of their kinase domains to other TKs (7, 8).

**Fig.1:**
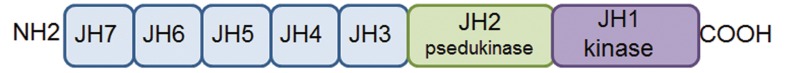
Schematic structure of Janus kinases (JAK). JH; JAK homology domain. Kinase domain is located in JH1. JH2 has pseudokinase activity.

JAK1 is a member of a new class of protein-TKs (PTKs) characterized by the presence of a second phosphotransferase related domain immediately N-terminal to the PTK domain. The second phosphotransferase domain bears all the hallmarks of a protein kinase, although its structure differs significantly from that of the PTK and threonine/serine kinase family members. JAK1 is a large, widely expressed membrane-associated phosphoprotein involved in the interferon-alpha/beta and -gamma signal transduction pathways. The reciprocal interdependence between JAK1 and TYK2 activities in the interferon-alpha pathway as well as between JAK1 and JAK2 in the interferon-gamma pathway may reflect a need for these kinases in the correct assembly of interferon receptor complexes. Binding of cytokines, growth factors and hormones to specific receptors leads to activation of various TKs. These kinases include JAKs, receptor TKs, and non-receptor TKs such as Src and ABL, which can directly phosphorylate STAT proteins without ligand-induced receptor signaling (9-11). They phosphorylate a tyrosine residue of STATs, followed by their dimerization through the reciprocal Src homology 2 (SH2)-phosphotyrosine interactions which lead to nuclear translocation and transcriptional activation of the target genes (12-15). The JAK protein are relatively large kinases with more than 1100 amino acids and apparent molecular weights of 120-130 kDa (Table 2). JAK has seven defined regions of homology called the Janus homology domain (JH) 1-7 (Fig .1). JH1 is a kinase domain important for JAK enzymatic activity where phosphorylation of its tyrosines leads to conformational changes in the JAK protein to facilitate substrate binding. JH2 is a pseudokinase domain, a domain structurally similar to a TK essential for normal kinase activity yet lacks enzymatic activity. The JH3-JH4 domains of JAKs share homology with SH2 domains. The amino terminal (NH_2_) end (JH4-JH7) of JAKs is called a FERM domain (short for band 4.1 ezrin, radixin and moesin); this domain is also found in the focal adhesion kinase (FAK) family and is involved in association of JAKs with cytokine receptors and/ or other kinases (16).

In summary it appears that specific JAK kinases, either alone or in combination with other JAKs, may be preferentially activated depending on the receptor that is being activated. Subsequentially different STATs will undergo activation.

**Table 2:** Characteristics of Janus kinase (JAK) and signal transducers and activators of transcription (STAT) members


Member	Chromosomal location	Isoform	Gene size (bp)	mRNA size (bp)	Amino acid	MW (KDa)

*JAK1*	1p32.3	-	133,282	5,053	1,154	130
*JAK2*	9p24	-	142,939	5,285	1,132	125
*JAK3*	19p13.1	-	23,251	5,449	1,124	115
*TYK2*	19p13.2	-	30,045	4,262	1,187	140
*STAT1*	2q32.2	Alpha*	45,215	4,326	750	91
		Beta	38,714	2,798	712	
*STAT2*	21q13.3	I	18,657	4,576	851	113
		II		4,564	847	
*STAT3*	17q21.31	I*	75,171	4978	770	80
		II		4,935	769	
		III	75063	4819	722	
*STAT4*	2q32.2-.3	-	121,620	2,761	784	81
*STAT5*	17q11.2	a	24,397	4,314	794	
		b	77,230	5,171	787	90
STAT6 **	12q13	I	16,010	4,050	874	
		II	10,668	3,755	737	
				3,894		
			11,707	3,976	847	90-110
				4,031		


*; Canonical active member,**; STAT6, has transcript variant in addition of its isoforms and MW; Molecular weight.

### STATs structure and activation

**Fig.2:**
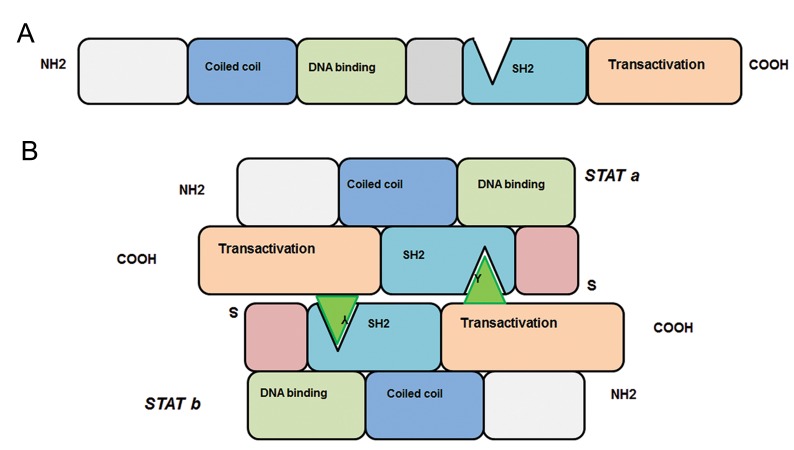
Schematic signal transducers and activators of transcription (STAT) structure. A. Inactive form of STAT monomer before C-terminal tyrosine (Y) phosphorylation and B. STAT dimerization and activation after C-terminal tyrosin (Y) phosphorylation (three angles) and bound to the SH2 domain of the other juxta STAT. SH2; Src homology 2 and NH2; Amino terminal.

**Fig.3:**
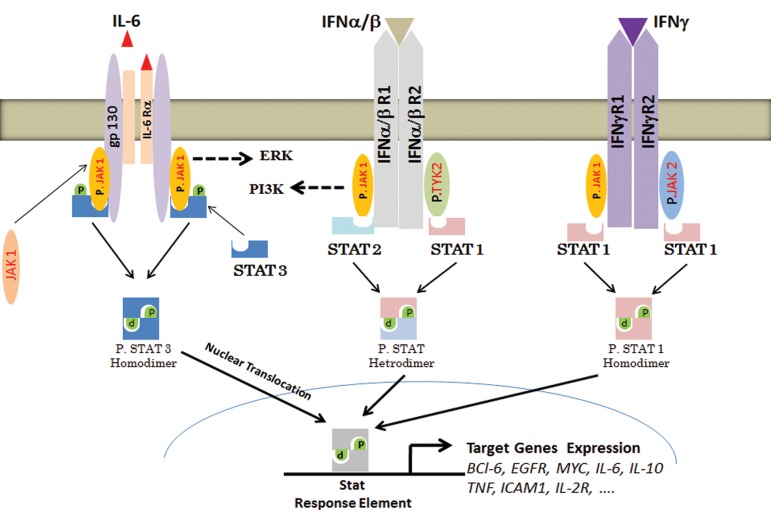
Cytokines induce Janus kinase/signal transducers and activators of transcription (JAK/STAT) pathway activation. Expression of STAT target gene is dependent on STAT types as well as cell types. IL; Interleukin, INF; Interferon, ERK; Extracellular regulated MAP kinase, PI3K; Phosphoinositide 3-kinase and TYK; Tyrosine kinases.

The seven mammalian STATs bear a conserved tyrosine residue (Y) near the C-terminus that is phosphorylated by JAKs. This phosphotyrosine allows for dimerization of a STAT (STATa) by a second STAT (STATb) through interaction with a conserved SH2 domain of the second STAT. Phosphotyrosine of the second STAT also interacts with the SH2 domain of STATa (Fig .2). Phosphorylated and dimerization of STATs will occur. The STAT dimer enters the nucleus where it binds specific regulatory sequences to activate or repress transcription of target genes by direct DNA binding (Fig .3) or by associating with other transcription factors (17, 18). The activity of STATs can be abolished by mutation of this critical tyrosine (19, 20). Each active homodimer STAT can induce the expressions of several target genes which are dependent upon both cell and STAT types. According to the Transcriptional Regulatory Element Database, some genes have more than one type of STAT transcription factor (Table 3). The target genes of heterodimer STAT are unclear however they may depend on random binding of STATa or STATb to DNA which induces expression of target genes. In addition to gene expression by STAT, alterations can occur through association with other transcription factors and cofactors regulated by other signaling pathways. Thus integrating input from many signaling pathways must be considered.

### JAK/STAT pathway

Activation of the JAK/STAT pathway occurs by binding of ligands to their receptors. These ligands can activate different JAKs and STATs (Table 1). In addition to JAKs other non-receptor TKs can be phosphorylated and activated by interaction between ligands and their receptors in the JAK/STAT pathway (Table 4). The JAK family (for mammals: JAK1, JAK2, JAK3 and TYK2) activates when two JAKs are brought into close proximity and trans-phosphorylation is allowed. Once activated, JAKs can phosphorylate additional targets which include both the receptors and their major substrates, the STATs (Fig .3). Subsequently, phosphorylated STATs are transported into the nucleus and modulate expressions of several genes. In normal cells, after modulating gene expression, *STATs* become dephosphorylated by tyrosine phosphatases and are thus free for subsequent rounds of stimulation (21).

### JAK/STAT pathway inhibitors

There are three major classes of negative regulators which inhibit JAK/STAT pathway. Signaling is also inhibited via two additional pathways.

Suppressor of cytokine signaling (SOCS) family members are STAT target genes that bind to receptors and block further STAT activation by turning off the initial signal (Table 5) (22).

**Table 3:** Human signal transducers and activators of transcription (STATs) target genes and gene chromosomal localization http://rulai.cshl.edu/cgi-bin/TRED/tred.cgi?process=home


STAT1 target genes							
Gene	Location	Gene	Location	Gene	Location	Gene	Location

*A2M*	12p13.3	*GATA3*	10p15	*PBF*	8p21.1	**REV3L	6q21
*APOE*	19q13.2	*GBP1*	1p22.2	*JUN*	1p32-31	*RNMT*	18p11.22
*B3GAT3*	11q12.3	*HSPB1*	7q11.23	*LTC4S*	5q35	*SEC6L1*	5p15.33
*BCL6*	3q27	*HSPCA*	14q32.33	*MAT2A*	2p11.2	*SOCS3*	17q25.3
*CASP4*	11q22.2	*ICAM1*	19p13.3	*MET*	7q31	*TAP1*	6p21.3
*CLC*	19q13.1	*IFNA1*	9p22	*MHC2TA*	16p13	*TIMP1*	Xp11.3
*CLC*	11q13.3	*IFNG*	12q14	*MUC1*	1q21	*TIMP3*	22q12.3
*CDKN1A*	6p21.2	*IL2RA*	10p15	*MYC*	8q24.12	*TLR2*	4q32
*CSF1*	1p21-13	*IL6ST*	5q11	*PIM1*	6p21.2	*TNFRSF5*	20q12
*CYP19A1*	15q21.1	*IRF1*	5q31.1	*PLAU*	10q24	*TNFRSF8*	1p36
*EGFR*	7p12	*IRF7*	11p15.5	*PRF1*	10q22	*TP53*	17p13.1
*FCGR1A*	1q21.2	*JAK3*	19p13.1	*PSMB9*	6p21.3	*VIP*	19p13.12
*FCGR3A*	1q23	*NOL3*	16q21	*PTGFR*	1p31.1	*VIP*	6q25
*FOS*	14q24.3	*NOS2A*	17q11.2	*REG1A*	2p12		

**STAT2 target genes**							

*APOE*	19q13.2
*RF7*	11p15.5

**STAT3 target genes**							

*A2M*	12p13.3	*FOS*	14q24.3	*MIA2*	14q13.2	*SOCS3*	17q25.3
*B3GAT3*	11q12.3	*HMOX1*	22q13.1	*MUC1*	1q21	*SOS1*	2p22-21
*BCL2*	18q21.3	*HSPCA*	14q32.33	*MUC4*	3q29	*STRA13*	17q25.3
*BCL2L1*	20q11.21	*HSPCB*	6p12	*MYC*	8q24.12	*TIMP1*	Xp11.3
*BIRC5*	17q25	*IGF1*	12q22-23	*NOL3*	16q21-23	*TIMP3*	22q12.3
*CCL2*	17q11.2	*IL10*	1q31-32	*NOS2A*	17q11.2	*TLR2*	4q32
*CCND1*	11q13	*IL2RA*	10p15-14	*OSM*	22q12.2	*TNF*	6p21.3
*CCND3*	6p21	*IL6*	7p21	*OXTR*	3p25	*TNFRSF5*	20q12
*CDKN1A*	6p21.2	*IL6ST*	5q11	*PBF*	8p21.1	*TNFRSF6*	10q24.1
*CEBPB*	20q13.1	*IRF1*	5q31.1	*PIM1*	6p21.2	*TNFRSF8*	1p36
*CSRP1*	1q32	*JAK3*	19p13.1	*PRF1*	10q22	*TRH*	3q13.3
*CYP19A1*	15q21.1	*JUN*	1p32-31	*REG1A*	2p12	*VEGF*	6p12
*EHHADH*	3q26.3-28	*KIAA0146*	8p11.2	*RORA*	15q21-22	*VIP*	6q25
*FASN*	17q25	*LBP*	20q11.23	*SEC6L1*	5p15.33	*VIP*	19p13.12
*FCGR1A*	1q21.2	*MCL1*	1q21	*SOCS1*	16p13.13		

**STAT4 target genes**							

*AICDA*	12p13	*IL2RA*	10p15-14	*MYC*	8q24.12	*PIM1*	6p21.2
*IFNG*	12q14	*IRF1*	5q31.1	*PBF*	8p21.1	*PRF1*	10q22

**STAT5 target genes**							

*ANGPTL4*	19p13.3	*CSN2*	4q21.1	*IL6ST*	5q11	*PRF1*	10q22
*BCL2*	18q21.3	*EGFR*	7p12	*MET*	7q31	*RARA*	17q21
*BCL2L1*	20q11.21	*ESR1*	6q25.1	*MUC1*	1q21	*RNMT*	18p11.22
*BCL6*	3q27	*ESR2*	14q	*OSM*	22q12.2	*SEC6L1*	5p15.33
*CCND1*	11q13	*IFNG*	12q14	*PAX5*	9p13	*TIMP3*	22q12.3
*CCND2*	12p13	*IGF1*	12q22-23	*PBF*	8p21.1	*TNF*	6p21.3
*CCND3*	6p21	*IL2RA*	10p15-	*PIM1*	6p21.2	*TNFRSF5*	20q12
*CEL*	9q34.3	*IL6*	7p21	*PPARG*	3p25	*TRIP15*	15q21.2

**STAT6 target genes**							

*ADAM8*	10q26.3	*CCL11*	17q21.1	*NCOA3*	20q12	*TNF*	6p21.3
*ADRA2B*	2p13	*IL1R1*	2q12	*PRKCA*	17q22	*TNFRSF5*	20q12
*ALOX15*	17p13.3	*IRF1*	5q31.1	*SELE*	1q22-25
*BCL2L1*	20q11.21	*IRF4*	6p25-23	*SOCS1*	16p13.13


**Table 4:** Janus kinase (JAK),signal transducers and activators of transcription (STAT) and other tyrosin kinases (TKs) are activated by several cytokines http://www.cellsignal.com/reference/pathway/jakstat_utilization.html


Ligand	Receptor	JAK	Other TKs	STAT family members

IL-6	IL-6Ra+gp130	JAK1, 2, TYK2	Hck	STAT1, STAT3
IL-11	IL-11R+gp130	JAK1, 2, TYK2	Src, Yes	STAT3
CNTF, CT-1, LIF, OSM	CNTFR+gp130, CT-1R+gp130, LIFR+gp130, OSMR+gp130	JAK1, 2, TYK2	Src family	Predominant: STAT3 Secondary: STAT1, 5
G-CSF	G-CSFR	JAK2, TYK2	Lyn	STAT3
IL-12 (p40+p35)	IL-12Rβ1+IL-12Rβ2	JAK2, TYK2	Lck	STAT4
Leptin	LeptinR	JAK2	NR	STAT3, 5, 6
IL-3	IL-3Rα+βc	JAK2	Fyn, Hck, Lyn	STAT3, 5, 6
IL-5	IL-5R+βc	JAK2	Btk	STAT3, 5, 6
GM-CSF	GM-CSFR+βc	JAK2	Hck, Lyn	STAT3, 5
Angiotensin	GPCR	JAK2, TYK2	NR	STAT1, 2, 3
Serotonin	GPCR	JAK2	NR	STAT3
α-Thrombin	GPCR	JAK2	NR	STAT1, 3
Chemokines	CXCR4	JAK2, 3	NR	NR
IL-2	IL-2Rα+IL-2Rb+γc	JAK1, 2, 3	Fyn, Hck, Lck, Syk, Tec	STAT3, 5
IL-4	IL-4Rα+γcR or IL-4Rα+IL-13Rα1	JAK1, 3	Lck, Tec	STAT6
IL-7	IL-7R+γc	JAK1, 3	Lyn	STAT3, 5
IL-9	IL-9R+γc	JAK1, 3	NR	STAT1, 3 ,5
IL-13	IL-13Rα1+IL-4Rα	JAK1, 2, TYK2	Ctk	STAT6
IL-15	IL-15Rα+IL-2Rβ+γc	JAK1, 3	Lck	STAT3, 5
IL-19	IL-20Rα+IL-20Rβ	JAK1, ?	NR	STAT3
IL-20	IL-20Rα+IL-20Rβ, IL-22R+IL-20Rβ	JAK1, ?	NR	STAT3
IL-21	IL-21R+γc	JAK1, 3	NR	STAT1, 3, 5
IL-22	IL-22R+IL-10Rβ	JAK1, TYK2	NR	STAT1, 3, 5
IL-23 (p40+p19)	IL-12Rβ1+IL-23R	JAK2	TYK2	STAT4
IL-24	Same as IL-20	JAK1, ?	NR	STAT3
IL-26	IL-20Rα+IL-10Rβ	JAK1, TYK2	NR	STAT3
IL-27 (EBI3+p28)	gp130+WSX1	JAK1, 2, TYK2	NR	STAT1, 2, 3, 4, 5
IL-28A, IL-28B, IL-29	IL-28R+IL-10Rβ	JAK1, TYK2	NR	STAT1, 2, 3, 4, 5
IL-31	IL-31Rα+OSMR	JAK1, 2, TYK2	NR	STAT1, 3, 5
IL-35 (p35+EBI3)	gp130+WSX1	JAK1, 2, TYK2	NR	STAT1, 3, 5
GH	GHR	JAK2	Src family	STAT3, 5(mainly STAT5a)
Tpo	TpoR (c-Mpl)	JAK2, TYK2	Lyn	STAT1, 3, 5
Epo, Pro	EpoR, ProlactinR	JAK2	Src family	STAT5 (mainly STAT5a)
Interferon (IFNα/β)	IFNAR1+IFNAR2	JAK1, TYK2	Lck	Predominant: STAT1, 2 Secondary: STAT3, 4, 5
IFN-γ	IFN-gR1+IFN-γR2	JAK1, JAK2	Hck, Lyn	STAT1
IL-10	IL-10Rα+IL-10Rβ	JAK1, TYK2	NR	STAT1, 3, 5
TLSP	TLSPR and IL-7R	JAK1, possibly JAK2	NR	STAT3, 5
EGF	EGFR	JAK1	EGFR, Src	STAT1, 3, 5
PDGF	PDGFR	JAK1, 2	PDGFR, Src	STAT1, 3, 5


NR; Not reported, bc; Common beta receptor subunit, gc; Common gamma receptor subunit, Epo; Erythropoietin receptor and Tpo; Thrombopoietin receptor.

**Table 5:** Suppressor of cytokine signaling (SOCSs) express by different cytokines and suppress the Janus kinase/signal transducers and activators of transcription (JAK/STAT) pathway by a negative feedback mechanism


Upregulator	SOCS member	Inhibit signal induced by

IL-6, IFNγ	SOCS1	IL-2, 3 ,4, 6, IFNα, IFNγ, GH, Epo
IL-2, 6, IFNα, IFNγ, GH	SOCS2	IL-6, GH, Epo
IL-6, IFNγ	SOCS3	IL-2, 3, 4, 6, IFNα, IFNγ, GH, Epo
NR	SOCS4	NR
NR	SOCS5	IL-6
NR	SOCS6	NR
NR	SOCS7	NR


IL; Interleukin, IFN; Interferon, NR; Not reported, GH; Growth hormone and Epo; Erythropoietin.

Protein inhibitors of activated STAT (PIAS) include PIAS1, PIAS2, PIAS3, PIAS4, PIASx and PIASy. These proteins have a Zn-binding ring-finger domain in the central portion. The PIAS proteins bind to activated STAT dimers and prevent them from binding DNA. PIAS1 and PIAS3 bind to STAT1 and STAT3, respectively. They inhibit transcriptional activity of the STATs, but do not affect phosphorylation. Just how specific they are in terms of regulating cytokine signaling remains to be determined; no knockouts have yet been reported (23).

Tyrosine phosphatases are the simplest way to reverse JAKs activity. The best characterized of these is the SH2 domain that contains protein tyrosine phosphatase-1 (SHP-1). It contains two SH2 domains and can bind to either phosphorylated JAKs or phosphorylated receptors to facilitate dephosphorylation of these activated signaling molecules.

SOCS proteins are a family of at least eight members that contain an SH2 domain and a SOCS box at the C-terminus. In addition, a small kinase inhibitory region located N-terminal to the SH2 domain has been identified for SOCS1 and SOCS3. The SOCS are responsible for a negative feedback loop in the JAK/STAT circuitry: activated STATs stimulate transcription of the SOCS genes. The resultant SOCS proteins bind phosphorylated JAKs and their receptors to turn off the pathway. SOCS can affect their negative regulation by three means: binding phosphortyrosines on the receptors (SOCS physically block the recruitment of signal transducers to the receptor), binding directly to JAKs, or to the receptors to specifically inhibit JAK kinase activity (Table 3) (24).

In addition to SOCS, PIAS and SHIP-1 that have negative regulatory roles in active STATs, sumoylation (small ubiquitin-like modifier) is another system that controls STAT activity, however its exact mechanism is not known. Thus, it will be important to characterize the physiologic function of this family of molecules (23).

Activation of STATs and JAKs can mediate the recruitment of other molecules involved in signal transduction such as the Src-family kinases, protein tyrosine phosphatases, Mitogen-activated protein kinase (MAP) kinases, and Phosphoinositide 3-kinase (PI3K) kinase. These molecules process downstream signals via the Ras-Raf-MAP kinase and PI3 kinase pathway which results in the activation of additional transcription factors. The combined action of STATs and other transcription factors activated by these pathways dictate the phenotype produced by a given cytokine, interferon stimulation (25, 26). STATs have also been shown to play roles in the inflammatory signaling cascades triggered by lipopolysaccharide (LPS), interferon gamma (INFγ) and other cytokines (27-30). STAT1 and STAT3 have been implicated as key transcription factors in both immunity and inflammatory pathways (31, 32). In addition, it has been shown that LPS-induced interleukin-1β (IL-1β) production in macrophages is in part regulated through JAK2. The STAT3 pathway is activated in response to several cytokines such as IL-1β, IL-4 and IL-10 (33, 34). Additionally, STAT3 has a dual role in IL-6 mediated signaling; its activation may result in increased IL-6, but also IL-6 itself may lead to phosphorylation of STAT3 which results in diverse biological responses (6, 35). The DNA binding region of STATs resides within the central 171 amino acids, but relatively few direct contacts exist. Rather, the clamp-like structure is imparted by phosphotyrosine-SH2 interactions. STATs bind two types of DNA motifs: IFN-stimulated response elements (consensus: AGTTTNCNTTTCC) and IFNγ-activated sequence elements (consensus: TTCNNNGAA). STAT1, STAT2, and p48 bind to IFN-stimulated response elements whereas STAT1, STAT3, STAT4, STAT5a, and STAT5b bind to IFNγ-activated sequence element sites. STAT6 binds a similar but distinct site: TTCNNNNGAA (36). STAT1, STAT2, and STAT5 contain carboxy-terminal transcriptional activation domains. It has been shown that STAT1, STAT3, STAT4, and STAT5 are phosphorylated on serine residues in response to cytokine stimulation. For these proteins, a conserved site of serine phosphorylation that remains in a consensus sequence for MAPK-mediated phosphorylation has been mapped within the carboxy-terminal transcriptional activation domain. However the functional significance of STAT serine phosphorylation and the identity of the kinase(s) responsible for this event are controversial. Recently, a large number of reports have been published that STAT serine phosphorylation to the activation of various MAPKs. Notably they provide significantly divergent results, perhaps due to the differences in the STAT proteins investigated and in the systems utilized (37-40).

According to a PubMed search, until today more than 17700 STATs papers have been published. Most have discussed the direct and indirect functions of STATs which show the important role of STATs in molecular cell biology. The numbers of publications are as follows: STAT3 (40.5%), STAT1 (25%), STAT5 (18%), STAT6 (8.6%), STAT4 (4.5%), and STAT2 (3.4%). The large number of STAT3 publications possibly show contribution of STAT3 in the JAK/STAT pathway compared to other STATs. Here we focus on the biology of STAT3 and briefly describe the roles of this STAT on hemostasis and malignancies, including hematopoietic disorders.

### STAT3

The protein encoded by this gene is a member of the STAT protein family. STAT3 is activated through phosphorylation in response to various cytokines and growth factors that include IFNs, EGF, IL5, IL6, HGF, LIF, IL-11, Ciliary neurotrophic factor (CNTF), Macrophage colony-stimulating factor 1 (CSF-1), Platelet-derived growth factor (PDGF), Oncostatin-M (OSM) and Bone morphogenetic protein 2 (BMP2) (Tables1, 4). This protein mediates the expression of a variety of genes in response to cell stimuli and thus plays a key role in many cellular processes such as cell growth and apoptosis. The small GTPase Rac1 has been shown to bind and regulate the activity of this protein. PIAS3 protein is a specific inhibitor of STAT3. Three alternatively spliced transcript variants that encode distinct isoforms have been described (Table 2). A number of factors regulate the JAK-STAT pathway including STAT dephosphorylation by phosphatases, altered nuclear import- export dynamics of STAT and STAT gene activation antagonists such as SOCS and PIAS (41, 42). STAT3 forms a homodimer or heterodimer with a related family member (at least STAT1). This molecule interacts with IL-31 receptor subunit alpha (IL31RA), Nuclear receptor coactivator 1 (NCOA1), Proline-, glutamic acidand leucinerich protein 1 (PELP1), Suppressor of cytokine signaling 7 (SOCS7), Hepatitis C (HCV) core protein and IL23R in presence of IL23. STAT3, via the SH2 domain, interacts with Serine/threonine-protein kinase NLK2 (NLK), Importin subunit alpha-3 (KPNA4), Importin subunit alpha-6 (KPNA5), Importin subunit alpha-3 (KPNA4), and Caveolin-2 (CAV2). It phosphorylates on serine region after DNA damage, probably by Serineprotein kinase ATM or Serine/threonine-protein kinase ATR. Serine phosphorylation is important for the formation of stable DNA-binding STAT3 homodimers and maximal transcriptional activity.

### STAT3 in development and differentiation

Among the mammalian STAT proteins, STAT3 is the most diverse in cell biology. Embryonic stem 405 (ES) cells can be maintained in an undifferentiated state by the addition of leukemia inhibitory factor (LIF) but expression of a dominant negative form of STAT3 leads to the differentiation of ES cells, even when LIF is present (43). 

Numerous cytokines induce expression of members of the anti-apoptotic regulator Bcl-2 family of proteins and STAT3 represses apoptosis in human myeloma cells by stimulating expression of Bcl-XL (44). 

T helper 17 (Th17) development from naive precursors is dependent upon signal transduction through STAT3. In mice, RORC is a STAT3 target gene and Th17 differentiation is induced by STAT3 signaling cytokines, notably IL-6, IL-21 and IL-23, which can be abrogated effectively by a deficiency in STAT3 (45). In humans, STAT3 deficiency from dominant negative mutations in the *STAT3* gene occur in hyperimmunoglobulin E recurrent infection syndrome (HIES or Job). This syndrome is characterized by morphological abnormalities, recurrent infections (particularly with Staphylococcus aureus and Candida sp.) and a deficiency of Th17 cells (46,48). Patients with HIES not only have reduced Th17 numbers, but their naive Th cells are resistant to Th17 differentiation under appropriate stimulatory conditions with concomitant impairment of RORγt expression relative to healthy controls. There are reasons to suspect that the STAT3/STAT5 signaling pathways are important in the conversion of regulatory T cells (Tregs) to Th17. First, there is evidence to suggest that STAT5 and STAT3 cross-regulate the conversion of naive T cells to Treg and Th17 lineages. This enables IL-6-activated STAT3 to inhibit both FoxP3 expression and enable IL-17 production in naive T cells stimulated with TGF-β (49). Not surprisingly, humans with HIES (who have mutations in STAT3) have a higher than normal percentage of cells that bear the phenotype of Tregs (50), while mice deficient in the IL-2 signaling cascade (notably IL-2 or STAT5) have a reduction in Tregs and an excess of Th17 cells in association with autoimmune disease. 

Granulocyte colony-stimulating factor (G-CSF) stimulates proliferation, survival, and differentiation of myeloid progenitor cells towards neutrophilic granulocytes (51). The biological effects of G-CSF are mediated through a cell surface receptor (G-CSF-R) of the hematopoietin or class I cytokine receptor superfamily (52). G-CSF activates STAT1, STAT3, and STAT5 (53). Whereas the membrane-proximal cytoplasmic region of the G-CSF-R is sufficient for activation of STAT1 and STAT5, activation of STAT3 requires the membrane-distal C-terminal part of the receptor (54). The G-CSF-R C-terminus contains four conserved tyrosine residues (Y704, Y729, Y744, and Y764) and comprises a region that has specifically been implicated in the control of neutrophilic differentiation (55). These tyrosines are also important for differentiation and survival signals from the G-CSF-R (56). According to another study, IL-6 and OSM-induced growth inhibition of A375 melanoma cells is dependent on STAT3 activation and correlates with increased transcript levels of the cdk inhibitor p27Kip1 (57). Finally Silver et al. (58) have reported that STAT3 is involved in G-CSF-mediated differentiation, survival and regulation of p27 Kip1 expression. In addition, it has shown perturbations in the proliferation/differentiation balance of myeloid progenitor cells of p27-deficient mice in response to G-CSF. Based on these data, STAT3-mediated expression of p27 is proposed to represent one of the mechanisms by which G-CSF controls differentiation and survival of myeloid progenitor cells (58). 

Inhibition of STAT3 activity in tumor-derived cell lines both *in vitro* and *in vivo*, by the introduction of antisense, small interfering RNA, decoy molecules, dominant negative STAT3 constructs, and/or blockade of TKs has been associated with growth arrest, apoptosis, decreased angiogenesis and invasion (59,61). More recently, non-canonical functions for STAT3 have been identified which include non-tyrosine phosphorylated STAT3 mediating transcriptional activation, non-tyrosine phosphorylated STAT3 binding to stathmin (a microtubule associated protein) and regulating migration, and nontyrosine phosphorylated STAT3 regulating metabolic functions in the mitochondria leading to Ras-dependent transformation (62,64). 

### STAT3 inducer and inhibitor agents

Numerous JAK/STAT inhibitory pathways are inactivated in cancer cells which results in constitutively activated STATs. In addition to the canonical role of STATs in regulating transcription, STAT3 has other non-transcription based roles. Tyrosine phosphorylated STAT3 may be located at the leading edge of migrating cells, specifically at focal adhesions, where it promotes migration (65). Both JAKs and STATs can be associated with microtubules (66), and the interaction between STAT3 and microtubules promotes migration by competing with binding the microtubule associated protein stathmin (67). STAT3 is activated in 70% of breast tumors and often associated with both aggressive and invasive tumors (68). Inhibition of STAT3 leads to a reversion of the malignant phenotype of these cells, which indicates that it is a key mediator of breast cancer pathogenesis. Elucidating the role of STAT3 in breast cancer and identifying methods to inhibit STAT3 can be of benefit for developing cancer treatments. Microtubule-targeting agents are among the most active drugs used as breast cancer treatment. Two types are utilized: microtubule stabilizers such as paclitaxel (Taxol) and microtubule destabilizers such as vinorelbine (Navelbine). Since STAT3 is activated in most breast cancers and associates with microtubules, Taub (69) have shown that microtubule-targeted therapy modulates STAT3 signaling and function in breast cancer cells. ObR is a single transmembrane protein that belongs to the class I cytokine receptor superfamily (9). Leptin binding induces activation of JAK2 and *STATs*, particularly STAT3 (10,70). Among the splicing variants of leptin receptors, only the long form ObRb induces STAT3 activation. 

### STAT3 signaling in malignancy

As one of the STAT family members, STAT3 is correlated with positive regulation of cell growth and highly activated in cancer cells (9,71). In cancers of epithelial origin, STAT3 is constitutively activated in head and neck squamous cell carcinoma (HNSCC) (72,73), breast cancer cell lines (74,75), ovarian cancer cell lines (76), lung cancer cell lines (77) and myeloma cell lines (14). In particular, STAT3 plays a critical role in the development of skin cancer (78). Activation of STAT3 signaling regulates the expression of numerous genes involved in growth control and survival. Studies have shown that numerous genes which encode for *BCL-XL, MCL-1, cyclins D1/D2*, and c-MYC proteins are downstream targets of STAT3 (7,17,79,80). Recent study has indicated that constitutive STAT3 signaling induces vascular endothelial growth factor (VEGF) expression and tumor angiogenesis (81). In positive feedback, the VEGF-VEGFR pathway leads to activation of STAT3 proteins and thus increases MCL-1 and XIAP (molecules involved in counteracting apoptosis) (82). The expression of VEGF antigen in gastric cancer cells can serve as a pertinent predictive factor for hematogenous invasion or metastasis; its importance has been proven and widely studied (83,87). In addition, the resistance to 5-fluorouracil (5-FU) is a main obstacle in gastric cancer chemotherapy. Dysregulation of STAT signaling pathways, particularly STAT3 and STAT5, has been demonstrated to contribute to malignant cellular transformation. STAT proteins are postulated to play important roles in oncogenesis by two distinct mechanisms: constitutive activity of the fulllength molecule and expression of a c-terminally mutated one. STAT proteins (in particular STAT3) are persistently in many cancer-derived cell lines (88,89). STAT3 is found to be constitutively phosphorylated to high levels in >50% of breast cancer derived cell lines, in >30% of breast adenocarcinomas and may be a poor prognostic indicator (90,91). Constitutive activation of STAT3 in epithelial cancers and cancer derived cell lines is frequently due to aberrant autocrine or paracrine IL-6 signaling (92). In myeloma cell IL-6 induced proliferation, activation of Src family kinases is required through CD45 molecules as well as activation of STAT3 and MAPK via the IL-6/IL-6 receptor complex (93). Mounting evidence gives credence to STAT3 as a critical mediator of oncogenesis that participates in human malignancies. Of human cancer, there is a high frequency of activation of STAT1, STAT3 and STAT5, with a higher incidence of abnormal STAT3 activation in most tumors studied. As the list of human tumors that harbor constitutive *STAT3* activity grows, there is an increasing chance that many more cases of human cancers will be identified in which STAT3 has a prominent role in induction and/or maintenance of the oncogenic phenotype. Constitutive STAT3 tyrosine or serine phosphorylation has been detected in breast carcinomas (94), HNSCC (95), as well as lymphomas and leukemias (96), as well as prostate, melanoma, pancreas, ovarian and brain tumors (33). STAT3 activates NFĸB in chronic lymphocytic leukemia (CLL). CD5 in CLL B cells controls IL-10 secretion through STAT3 and Nuclear factor of activated T-cells 2 (NFAT2) activation (97,98). c-ABL regulates *MCL-1* gene expression (a major target of STAT3) and recent studies show that STAT3 phosphorylation in CLL cells is dependent on c-ABL activity (99). These observations make it compelling to examine the role of STAT3 signaling in malignant progression in order to establish whether the constitutive STAT3 activation present in human tumors is essential for malignancy. The cancer-causing propensity of constitutively activated STAT3 protein and the evidence of potential clinical benefits of blocking constitutive STAT3 signaling make strong arguments for target validity of STAT3 for drug intervention in cancer therapy. JSI-124 (cucurbitacin I), a STAT3 inhibitor, decreases anti-apoptotic protein XIAP expression and potently induces cell-cycle arrest with subsequent apoptosis in some B-leukemia cell lines (100). STX-0119 (inhibitor of STAT3 dimerization) shows strong growth-inhibitory activity through apoptosis and down-regulation of STAT3 targets such as c-MYC, cyclin D1, Survivin and Bcl-xL (101). The obvious final question is whether oncogenesis can be induced in a STAT3 null genetic background by oncoproteins such as v-Src that induce STAT3 signaling. Gene knockout approaches do not lend themselves readily to biological studies of STAT3 signaling for the reason that early attempts to create STAT3 knockout mice have led to embryonic lethality at days 6.5 -7.5, an observation consistent with a biological role for STAT3 as mediator of self-renewal (36) and its absolute requirement for development, growth and survival. Recent efforts have generated conditional STAT3 knockouts (102), which will allow addressing the question of whether STAT3 null cells are indeed resistant to transformation by Src oncoproteins. 

## Discussion

STAT3 is a vital transcription factor activated by some ligands and IL-6 (103). It has important roles in mutagenesis and anti-apoptosis. STAT3 is involved in the transcriptional upregulation of many genes, not only acting by direct DNA binding, but in some cases as a coactivator of transcription factors such as activator protein-1 and hepatocyte nuclear factor-1 (104). STAT3 knockout results in early embryonic lethality, but conditional knockouts provide useful tools to examine the actions of STAT3 in specific tissues. In a study by Haga et al. (67), two animal models have been used to examine the effects of STAT3 modulation in Fas-mediated liver injury: mice injected with adenoviruses that expressed constitutively active STAT3 and other proteins, and mice with hepatocyte specific *STAT3* gene deletions. Intravenously injected adenoviruses normally home to the liver with infection of more than 80% of hepatocytes and allowing for expression of encoded proteins. Chan et al. (105) have demonstrated that constitutively active STAT3 provides protection against Fas-mediated liver injury and that STAT3 deficiency leads to Fas sensitivity. The anti-apoptotic proteins FLIP, Bcl-2, and BCL-XL block caspase activation and are elevated in IL-6–treated livers (106). Ng et al. (60) report elevations in these proteins in STAT3-overexpressing livers, which provides evidence that STAT3 mediates the major anti-apoptotic effects of IL-6. IL-6–mediated elevation of anti-apoptotic proteins is largely posttranscriptional, however mRNA for these proteins is elevated in the STAT3-overexpressing livers. This difference may be due to the massive overexpression of STAT3. Adenovirus infection confers a degree of transcriptional induction not seen in normal mice (106). As a result, STAT3 has been correlated with positive regulation of cell growth and is highly activated in cancer cells. miRNAs have a crucial function in oncogenesis by regulating cell proliferation and apoptosis as oncogenes or tumor suppressors. They play an important role in regulating various cell activities. miRNAs as small, non-coding, endogenous RNAs which regulate gene expression at the post-transcriptional level can be considered new therapeutic approaches. miRNAs are likely to be involved in most biologic processes by targeting signaling pathways. miR-21 which is abundantly expressed in various tumor cells, is a direct STAT3 target in Sezary cells (107). Up-regulation of miR-21 depends on activation of the ErbB/STAT3 pathway (108). INF induces miR-21 expression through STAT3 which directly binds the miR-21 promoter in response to IFN signaling. PTEN and AKT are downstream targets of miR-21 (109). IL-6 also activates STAT3 causing direct activation of miR-21 and miR-18b-1, which respectively inhibit PTEN and CYLD (tumor suppressor genes) (110). It is possible that the IL-6 anti-apoptotic pathway is linked to miRNA-21 (111). Overexpression of miR-155 (a putative oncomiR) leads to activation of STAT3. INF-γ and IL-6 upregulate expression of this miRNA (112). IL-10 is a potent anti-inflammatory cytokine that inhibits miR-155 transcription from the *BIC* gene through STAT3 (113). miR-21 is a key regulator of IL-11 signaling (114). miR-20b leads to downregulation of VEGF in breast cancer cells in a STAT3 dependent manner (115). Also miR-125b, miR-17, miR-20a and miR-106b target *STAT3* gene (116,117). One study has shown that miR-17-5p, miR-20a, miR-93, and miR-106a regulate STAT3 mRNA *in vitro* (118). miR-9 downregulates expression of JAK kinases and inhibits activation of STAT3 (119). miR-205 also inhibits STAT3 activation (120). Studies show that miR-199a and STAT3 are directly related (121). Given the wide role of STAT3 in cancer, miRNAs can be potentially considered as new therapeutic approaches for future research. 

In the past several years, compelling evidence has accumulated that highlights the role of STAT proteins in leukemogenesis. Constitutive activation of STATs has now been clearly demonstrated in acute and chronic leukemias. Elevated STAT3 activity has been observed in many spontaneous and experimentally established mammalian cancers, which demonstrates its critical role in tumorigenesis. Assessment of miRNAs expression patterns after the use of anticancer drugs can more precisely identify the molecular mechanisms of cancer cells. 
